# Process evaluation of the response of nursing homes to the implementation of the dementia-specific case conference concept WELCOME-IdA: A qualitative study

**DOI:** 10.1186/s12912-020-0403-6

**Published:** 2020-02-17

**Authors:** Daniela Holle, Sonja Teupen, Rabea Graf, Rene Müller-Widmer, Sven Reuther, Margareta Halek, Martina Roes

**Affiliations:** 1 Department of Nursing Science, University of Applied Sciences (hsg Bochum), Gesundheitscampus 6-8, 44801 Bochum, Germany; 2 German Center for Neurodegenerative Diseases (DZNE), Stockumer Str. 12, 58453 Witten, Germany; 3 Faculty of Health, University of Witten/Herdecke, Alfred-Herrhausen-Straße 50, 58455 Witten, Germany; 4 Städtische Seniorenheime Krefeld, De – Greiff – Straße 194, 47803 Krefeld, Germany

**Keywords:** Dementia-specific case conference, Behavioural and psychological symptoms, Process evaluation, Nursing home, Dementia, Qualitative study, WELCOME-IdA

## Abstract

**Background:**

The implementation of clearly structured dementia-specific case conferences could be an important tool to enable nursing staff to properly analyse and manage challenging behaviour in nursing home residents with dementia. A process evaluation of the responses of nursing homes to the implementation of WELCOME-IdA (*Wittener model of case conferences for people with dementia – the Innovative dementia-oriented Assessment tool)* was carried out to gain insight into which key elements of the intervention were adopted by the nursing homes and which elements were adapted.

**Methods:**

This study was part of a larger process evaluation using a qualitative design. Thirty-four semi-structured telephone interviews and 15 focus group interviews were conducted in four nursing homes. The interviews were analysed using deductive content analysis, although inductive categories have been developed.

**Results:**

Nursing home staff adopted the roles of moderator, case reporter, keeper of the minutes and reflection partner in WELCOME-IdA, but the selection of the staff members who filled these roles differed across nursing homes. The recommended group size of 5–8 participants per case conference was sometimes adopted. The key element of having core nursing teams who participated continuously in all case conferences was not adopted at all. Instead, there was a high level of rotation among staff members. The pre-defined process structure of WELCOME-IdA was adapted in such a way that the assessment of the residents’ behaviour and the selection of the relevant domain for the behaviour analysis were conducted in advance of the case conference. The evaluation of the interventions was also organized differently.

**Conclusion:**

The scope of the response implies that WELCOME-IdA requires further adaptation to the requirements of nursing processes in nursing homes. The results provide important information on the selection of role keepers and offer insights into a) how knowledge of the structured training was circulated and transformed into self-organized case conferences and b) how knowledge was circulated throughout the entire processing of one case. Thus, these results can be used to optimize intervention and implementation. Overall, the intervention should allow more possibilities for tailored adaptation than it currently does.

## Background

Up to 82% of nursing home residents with dementia develop at least one neuropsychiatric symptom during the course of their disease [1, 2]; such symptoms are also referred to as challenging behaviours [3]. Behaviours such as screaming, wandering, apathy, depression or aggression are considered challenging if they pose challenges for nursing staff and other residents or the people with dementia themselves [3]. Challenging behaviour is associated with negative outcomes for people with dementia, such as decreased quality of life [4] and enhanced use of psychotropic medication [5], as well as distress for formal carers [6, 7]. Research indicates that challenging behaviour expresses the distress or suffering of the person with dementia and can therefore be seen as the expression of a physiological or psychological need [8]. Challenging behaviour can be caused by diverse biological, psychological and social factors that are specific to the person with dementia [8]. Understanding these underlying causal mechanisms is a prerequisite for managing challenging behaviour. Approaches are needed that support professional carers and care teams not only in analysing the multitude of causes of challenging behaviour but also in developing individual interventions based on the specific situation of the person with dementia [9].

The development of an analysis-focused approach to challenging behaviour is a complex process, and new tools and guidance are required to apply such approaches in daily practice. A dementia-specific case conference (DSCC) is an important tool for enabling nursing staff to analyse the various triggers influencing the challenging behaviour of residents with dementia [10]. The DSCC provides a method for structured reflection, which enhances learning at work and helps nursing staff cope with problematic situations such as challenging behaviours [11, 12]. The DSCC concept WELCOME-IdA (*Wittener model of case conferences for people with dementia – the Innovative dementia-oriented Assessment tool*) was developed based on a literature review [13], consultation with experts in the field of DSCCs [12] and the results of an initial feasibility study of the experiences of nursing staff and the factors that promote or inhibit the use of DSCCs for nursing home residents with dementia [10]. The effectiveness of WELCOME-IdA was investigated in a stepped-wedge cluster randomized controlled trial, FallDem [14]. During the study, WELCOME-IdA was applied in four nursing homes in Germany.

Implementing complex interventions such as WELCOME-IdA is demanding [15], and often, the actual uptake of a complex intervention in health care practice is hindered by diverse factors [16, 17]. Thus, to evaluate complex interventions, it is increasingly recommended to perform a process evaluation coupled with an effectiveness study that investigates how the intervention is applied to gain insights into the implementation components while conducting cluster randomized controlled trials [18, 19]. The results of such process evaluations can provide information on how an intervention might be reproduced in a specific health care context [15, 18]. Following these recommendations, a process evaluation was conducted in parallel to the FallDem trial [20]. The design and procedures of this process evaluation were based on a framework for process evaluations in cluster randomized trials of complex interventions developed by Grant et al. [21]. This framework comprises several key aspects of process evaluations. One of these key aspects is the response of clusters to the complex WELCOME-IdA intervention. The response of the cluster describes how the intervention was adopted by the participating nursing homes (cluster) and whether it was adapted during its implementation in the specific health care context [21]. Thus, the research question of this study was as follows:

*Which key elements of WELCOME-IdA were adopted by the nursing homes, and which were adapted by them?*

## Methods

### Study design

A mixed longitudinal and retrospective qualitative process evaluation design was used to answer the research question [22]. The study was part of a larger process evaluation of the FallDem trial [20, 23] and was conducted alongside the FallDem trial [14]. This report is based on the recommendations of the Consolidated Criteria for Reporting Qualitative Research (COREQ) (Additional file 1) [24].

### Intervention

WELCOME-IdA is defined as a structured, goal-directed, intra-professional method in which nursing staff are guided through the process of searching for potential triggers and causes of challenging behaviour with the help of a structured assessment instrument called the IdA (Innovative dementia-oriented Assessment System) (Additional file 2) [12]. The IdA was developed based on a comprehensive literature review [25] and expert consultation. A detailed description of the development and evaluation of IdA has been published elsewhere [26]. We chose an intra-professional nursing approach to WELCOME-IdA due to the results of a prior feasibility study [10]. Additional disciplines were not directly included in the DSCCs. The main reason was that physicians and therapists are not employed by nursing homes in the national health care system. Due to this structure, the direct participation of other professions in regular case conferences is scarcely feasible. At the same time, we decided not to involve residents or residents’ families in the DSCCs. The results of the feasibility study indicated that nursing staff did not feel competent to include residents’ relatives directly into the case conference. The shared decision-making process took place among the nursing staff. However, interdisciplinary consultation as well as the involvement of residents’ relatives in WELCOME-IdA took place before or after the DSCC.

**Fig. 1:**
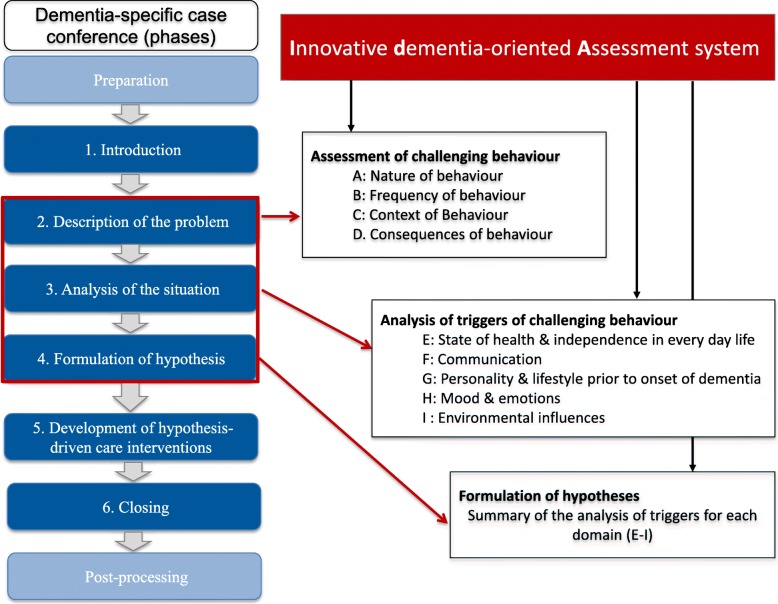
Process structure of WELCOME-IdA

WELCOME-IdA is embedded within the general theory of hermeneutics and the need-driven dementia-compromised behaviour (NDB) model [27, 28]. It includes a *predefined role structure* and a *process structure* (Fig. 1). Both help systemize reflection on the case (the resident with challenging behaviour) and prevent nursing staff from digressing in everyday conversations and from drawing hasty conclusions or developing poorly considered action plans during the DSCC.

The *role structure* includes four roles. (A) *Moderator*: The moderator is a person trained in moderating the case conference. She/he ensures adherence to method, time frame and roles. Adhering to the method involves process control, working through the different phases of WELCOME-IdA, and summary and visualization (e.g., using a flipchart) of the results. Adhering to the timeframe requires the moderator to ensure that sufficient time is available for the individual phases of WELCOME-IdA so that the DSCC can be completed on time. The moderator also ensures that the participants adhere to their assigned roles and tasks during the DSCC. (B) *Case reporter:* The case reporter prepares the necessary information for the DSCC. Based on this information, she/he presents the initial problem to the group and defines his or her expectations for the DSCC. The case reporter should be the primary caregiver of the discussed resident. She/he also ensures that the care interventions agreed upon in the DSCC are subsequently integrated into daily nursing practice. (C) *Keeper of the minutes:* The keeper of the minutes completes the IdA and writes down the central results in a protocol. (D) *Reflection partner*: A total of 2–5 people (core team members) should take on the role of reflection partners, who answer the IdA questions by gathering and supplementing information. They provide critical but supportive feedback, form hypotheses and develop hypothesis-driven care interventions.

WELCOME-IdA suggests that all roles in the DSCC except the moderator should be performed by people from the specific nursing ward where the case resident lives.

The *process structure* of WELCOME-IdA covers six phases: (1) introduction of the case, (2) description of the problem, (3) analysis of the situation, (4) formulation of hypotheses, (5) development and definition of hypothesis-driven care interventions and (6) closing. The IdA supports phases 2–4. For the description of the case, the IdA provides 14 guiding questions for assessing the challenging behaviour; these questions refer to the nature, frequency, context and consequences of the behaviour. For the analysis of the challenging situation, the IdA contains five different domains (state of health and independence in everyday life, communication, personality and lifestyle prior to the onset of dementia, moods and emotions, and environmental influences). Each domain ends with a summary of the results of the behaviour analysis, which supports the formulation of hypotheses about the possible triggers of the challenging behaviour (Fig. 1).

The DSCC should last between 60 and 90 min and should be conducted at least once a month, preferably in a room where there will be no disturbances.

Each DSCC should be preceded by a preparatory phase, which encompasses the *formal preparation* for the DSCC, *the selection of the case* and the *content-related preparation of the case.* Formal preparation primarily includes inviting the participants of the DSCC to the meeting and recording the times in the duty roster. Any team member can propose a resident with challenging behaviour as a case; the team jointly decides whether a DSCC is to be conducted. The proposing person then arranges the DSCC together with the leading ward nurse. A room must be available for the undisturbed completion of the DSCC. IdA sheets must also be prepared for the moderator and the keeper of the minutes.

The case reporter, which is usually the primary caregiver of the selected resident, conducts content-related preparation. Content-related preparation requires the case reporter to outline, at minimum, the problem to be discussed and his/her expectations for the DSCC. To increase the effectiveness of the DSCC, it is further recommended that background information on the resident concerned be collected and prepared.

The DSCC ends with a post-processing phase in which the entire nursing team is informed about the results of the DSCC. Furthermore, the results are documented in the nursing record. The agreed-upon care interventions are delivered to the resident and evaluated at the beginning of the next DSCC.

In each of the 4 nursing homes in the FallDem trial, the intervention started with 2 days of in-service training on WELCOME-IdA and was followed by four facilitated DSCCs per participating nursing team (on-the-job training). Subsequently, each nursing team was expected to conduct a minimum of four case conferences without any external assistance (off-the-job training). Additional training in moderation techniques was offered to at least 4 selected people (a group of moderators) per nursing home. Each nursing home was expected to establish a steering group. This group was responsible for the implementation process (such as the designation of responsibilities and the provision of structural requirements). The steering group was also in charge of conducting an assessment (at the beginning of the study) of the strengths and weaknesses of its organization in relation to the context in which the case conferences were conducted. Based on the results of this assessment, a tailored implementation plan was expected to be developed by the steering groups for each nursing home.

### Setting and sample

A total of 4 nursing homes with 7 nursing teams were recruited. Nursing homes were included if they were located in North Rhine-Westphalia due to the foundation’s purpose. The leading managers of the nursing homes had to select at least two nursing wards with two nursing teams to participate in the intervention study (Table 1). Three nursing homes belonged to a non-profit care provider; 1 nursing home belonged to a public provider. The nursing homes had a mean size of 78.3 residents (min. 54; max. 100), which is above the national average of 63 residents [29]. The nursing homes had a minimum of 2 units. Most residents had low to moderate levels of care dependency according to the Medical Service of the Health Insurance Funds (MDK) assessment. The levels of care dependency are consistent with the average national distribution of care levels in nursing homes [29]. On average, there were 51.8 care staff at each of the four nursing homes, but nursing home E82 had the lowest number of nursing staff (*n* = 38). Fifty percent of the nursing staff were registered nurses (qualified by a 3-year vocational training programme), which complies with the legal regulations for German nursing homes [30].

**Table 1:** Structural characteristics of the nursing homes at baseline

Nursing homes enrolled at baseline	Nursing homes			
	E29	E79	E75	E82
Nursing home size [n]	79	100	80	54
Number of units [n]	3	4	2	2
Residents´ level of care dependency [%]				
0	3.8	/	/	/
1 (low)	29.1	36.0	42.5	42.6
2 (moderate)	35.4	25.0	30.0	42.6
3 (severe)	29.1	33.0	25.0	9.3
3+ (very severe)	2.5	6.0	1.3	/
Number of total nursing staff [n]	51	59	60	38
Number of registered nurses* [n]	29	30	21	17
New employees, last 3 months	2	1	1	2
Employees who resigned, last 3 months	1	1	0	MD

Key: *n* number, *MD* missing data

None of the nursing homes had experience with WELCOME-IdA. All participants in the qualitative study were recruited via the stepped-wedge cluster randomized controlled trial (RCT) studying the effectiveness of WELCOME-IdA [14]. Each nursing home had a study coordinator who was responsible for recruiting the interview partners. All interview partners were provided with written material in advance of the interviews. For the telephone interviews, the participants gave verbal informed consent prior to each interview, which was then audiotaped. Written informed consent was obtained for all group interviews prior to data collection. None of the participants received financial incentives or gifts.

### Delivery of and participants in the intervention components

During the stepped-wedge cluster RCT conducted between September 2013 and March 2015, an average of 13.8 people (5–22) participated in the 1st WELCOME-IdA in-service training session, and 14.8 people (6–25) participated in the 2nd WELCOME-IdA in-service training session. They represented a skill mix of registered nurses and nursing assistants. The registered nurses comprised the largest group of participants in all nursing homes. Almost all people selected to be trained as moderators of DSCCs also attended the WELCOME-IdA in-service training sessions (Table 2) [23].

**Table 2:** Participants in the intervention components

Phase	Nursing homes						
	E29		E79		E75		E82
	n [MOD]		n [MOD]		n [MOD]		n [MOD]
1st WELCOME-IdA in-service training	19 [6]		22 [8]		9 [5]		5 [3]
2nd WELCOME-IdA in-service training	17 [6]		25 [9]		11 [5]		6 [4]
	Unit 1	Unit 2	Unit 1	Unit 2	Unit 1	Unit 2	Unit 1
	n [MOD]	n [MOD]	n [MOD]	n [MOD]	n [MOD]	n [MOD]	n [MOD]
1st DSCC on-the-job training	8 [5]	11 [6]	14 [4]	MD	9 [5]	8 [4]	4 [3]
2nd DSCC on-the-job training	10 [6]	12 [6]	16 [6]	MD	6 [3]	9 [4]	6 [1]
3rd DSCC on-the-job training	11 [6]	11 [6]	9 [5]	MD	7 [4]	7 [4]	4 [1]
4th DSCC on-the-job training	9 [6]	10 [6]	17 [4]	8 [1]	6 [4]	6 [4]	4 [1]
1st DSCC off-the-job training	15 [5]	12 [5]	–	–	MD	–	–
2nd DSCC off-the-job training	15 [4]	11 [5]	10 [3]	7 [3]	–	MD	–
3rd DSCC off-the-job training	19 [6]	17 [6]	10 [4]	8 [2]	–	MD	MD
4th DSCC off-the-job training	14 [5]	15 [5]	12 [2]	7 [1]	MD	–	–

Key: *MD* missing data, *MOD* number of skilled moderators, *DSCC* dementia-specific case conference, WELCOME-IdA = Wittener model of case conferences for people with dementia – the innovative dementia-oriented assessment tool, *n* number

WELCOME-IdA recommends that core nursing teams of 5–8 people participate in DSCCs, and this recommendation was partially observed during the WELCOME-IdA on-the job training and off-the-job training phases. Comparing the absolute number of participants (N) with the number who participated continuously (core team) in the intervention, only cluster E29 demonstrated the continuous participation of at least five core members. The people who continuously participated in all components of the intervention were mostly skilled moderators [23] (Table 2). The four nursing homes completed 47 DSCCs in total. Each nursing team performed 7 DSCCs on average (min 5; max 8) during the 7-month intervention phase (Table 2).

### Data collection

The data collection process consisted of longitudinal *semi-structured telephone interviews* and retrospective *focus group interviews*, all of which complemented one another to present a more comprehensive picture of the different objects under investigation. The telephone interviews aimed to obtain individual interviewees’ perspective on the ongoing implementation of the intervention, while the focus groups were conducted to learn how the different groups experienced the implementation process. Within both formats, the interviewees were addressed both as representatives of their organization as a group and as individuals. However, the dynamics of the group interviews resulted in data that can be understood as representing the participants’ individual perspectives on the organization.

*Telephone interviews:* A total of 34 structured telephone interviews with open questions [31] were conducted in parallel to the implementation of WELCOME-IdA (Table 3). The interviews aimed to assess whether the structured preparation and post-processing of the DSCCs had taken place and whether any adaptations of WELCOME-IdA occurred during its application in practice [20] (Table 4).

**Table 3:** Sequence of intervention phase and data collection per participating unit

Sequence of the intervention phase and data collection	
1st DSCC with support (on-the-job training)	
1st telephone interview	
2nd DSCC with support (on-the-job training) 3rd DSCC with support (on-the-job training)	
2nd telephone interview	
4th DSCC with support (on-the-job training) 1st DSCC without support (off-the-job training)	
3rd telephone interview	
2nd DSCC without support (off-the-job training)	
3rd DSCC without support (off-the-job training)	
4th telephone interview	
4th DSCC without support (off-the-job training)	
Focus group interviews with steering groups, moderators, and nursing teams	

Key: DSCC dementia-specific case conference.

**Table 4:** Characteristics of the interviewees in the telephone and group interviews

Interviewee	Telephone interviews	Group interviews		
	Nursing ward nurse/nurse manager (*n* = 34)	Steering groups (*n* = 4)	Moderators (*n* = 4)	Nursing teams (*n* = 7)
Total number of interviewees	9	48	33	65
Gender				
*Female*	7	31	28	56
*Male*	2	15	5	8
*No data*	–	2	–	1
Age [mean, (SD)]	40 (5.6)	45.3 (10.3)	41.3 (9.7)	43 (11.5)
Vocational education				
*Geriatric nurse (3-year education)*	8	23	22	19
*Nurse (3-year education)*	1	15	5	4
*Nursing assistant*	–	1	1	8
*Nursing assistant (no education)*	–	0	1	16
*Other*	–	7	4	16
*No data*	–	2	–	2
Working years [mean (SD)]	16.7 (5.9)	20.8 (9.9)	14.2 (8.3)	10.2 (8.6)
Workload				
*Full-time-job (100%)*	9	37	24	30
*Part-time job (> 50%)*	–	8	9	18
*Part-time job (< 50%)*	–	1	–	14
*No data*	–	2	–	3
Employed in nursing home since [mean (SD)]	11.8 (7.4)	9.8 (8.1)	7.1 (5.9)	6.2 (6.8)

Key: *SD* standard deviation, *n* number

The interviews were conducted promptly after the DSCCs were carried out, while the nurses’ memories of the previous DSCC and their preparations for it were still very present and information could be gathered regarding preparation for the upcoming DSCC. The telephone interviews were also a means to stay in close contact with the nursing teams.

The research team developed an interview guideline. RG, TQ, DH, UR, or SR performed all the phone interviews with 1 registered nurse per nursing team. For 6 of the 7 nursing teams, the selected nurse was the head nurse of the nursing team. For one nursing team, the interviews were conducted with the nurse manager (Table 4). The interviews lasted 15 min on average (min. 7; max. 24) and were audio recorded.

*Focus group interviews:* Fifteen semi-structured focus group interviews were conducted with the steering groups (n = 4), the moderators (n = 4) and the nursing teams (n = 7) at the end of the intervention phase (Table 1) to gain insight into the overall response of each nursing home to the intervention (Table 5). The research team created the semi-structured interview guideline. MH and MR performed all the interviews in the nursing homes. Field notes were taken during the interviews. The interviews lasted 40 min on average (min 25, max 61) and were audio recorded.

**Table 5:** Themes of semi-structured interview guidelines

Semi-structured telephone interviews with registered (head) nurses	Focus group interviews with		
	Moderators	Steering groups	Nursing teams
Preparatory phase of the DSCC Post-procession phase of the DSCC Appraisal of past DSCCs Implementation of the DSCC	Application of the DSCC Overall appraisal of the DSCC Implementation of DSCC Compilation of moderator team	Overall appraisal of the DSCC Implementation of the DSCC Compilation of the moderator team, steering group and core nursing teams	Application of the DSCC Overall appraisal of the DSCC Implementation of the DSCC

Key: *DSCC* dementia-specific case conference

### Data analysis

All interviews were transcribed verbatim and analysed using primarily deductive content analysis, although inductive categories have been developed [32]. This method focuses on the identification and categorical structuring of themes that emerge from the interviews. The main categories were derived deductively from the interview guidelines. The WELCOME-IdA manual [33] was used to develop a coding tree and to define the main categories in a code book (Additional file 3). The categories were further developed and differentiated inductively with reference to the data [32]. The coding was performed in sections (i.e., mostly based on a question and the participant’s answer) to make textual context available for the summary of the results. The coding was performed by one researcher (RG or DH) and checked by a second researcher (DH or ST) to improve inter-subjectivity and comprehensibility. All interviews were conducted and analysed in German. For the purpose of reporting the findings, individual German quotations were translated into English and proofread by a native English speaker to ensure the transparency and trustworthiness of the results [24]. The software MAXQDA 2018 was used for data analysis and data management.

## Results

In line with the key characteristics of WELCOME-IdA, the analysis revealed 4 separate but related themes: (1) response to role structure, (2) response to group size, (3) response to core nursing teams, and (4) response to process structure. The results are described in detail in the following. Table 6 summarizes the main results.

**Table 6:** Overview of the central findings related to the key characteristics of WELCOME-IdA

WELCOME-IDA	Response
Role structure	
*Moderator*	
− Internal or external person	− Often external moderator
− Training in moderating the DSCC	− Training in moderating the DSCC, regular peer debriefing
− Task: Ensure adherence to the method	− Task: Ensure adherence to method, e.g., by defining a certain seating arrangement
− Task: Ensure adherence to time frame	− Task: Ensure adherence to the time frame (partly performed by an additional co-moderator)
− Task: Ensure adherence to roles	− Task: Ensure adherence to roles and define rules for temporarily stepping out of one’s role
	− Task: Gain experience in each role and be a reference person for less experienced colleagues (peer support)
*Case reporter*	
− Internal person, primary caregiver of the resident	− Internal person, both the primary caregiver and a trained moderator
− Task: Prepare necessary case information	− Task: Prepare necessary case information, partly performed by two persons in tandem
− Task: Present the initial problem and define expectations for the DSCC	− Task: Present the initial problem and define expectations for the DSCC; process all IdA domains (a-e) in preparation for the DSCC and pre-select domain(s) for the behaviour analysis
− Task: Ensure subsequent integration of agreed upon care interventions into daily nursing practice	− Task: Ensure subsequent integration of agreed-upon care interventions into daily nursing practice (partly performed by a trained moderator/leading ward nurse)
*Keeper of the minutes*	
− Internal person	− Both an internal person and external person
− Task: Complete the IdA during the DSCC	− Task: Complete the IdA during the DSCC; in case of previously completed domains, document solely changes resulting from the discussion
− Task: Document central results in a protocol	− Task: Document central results in a protocol, partly with the collaboration of additional keepers of the minutes
*Reflection partner*	
− 2–5 internal persons (core team members)	− Different persons, no core team, a mixture of nursing staff from different wards, inclusion of social service staff
− Tasks: Answer the IdA questions by gathering and supplementing information, provide critical but supportive feedback, form hypotheses and develop hypothesis-driven care interventions	− Tasks: Answer the IdA questions by gathering and supplementing information, provide critical but supportive feedback, form hypotheses and develop hypothesis-driven care interventions
Group size	
− 5–8 participants	− Expanded group size to include more staff members, reduced group size due to small size of facility
Core nursing teams	
− 2–5 people should continuously participate in every DSCC	− No core nursing team due to several reasons: ad hoc selection of DSCC participants; aim to include more staff members; small size of facility; and absence of staff due to vacation, illness, and part-time employment
Process structure	
*Formal preparation for the DSCC*	
− Invitation of DSCC participants	− Invitation of DSCC participants, planning compensatory staff
− Recording of time and date in the duty roster	− Recording of time and date in the duty roster early
*Selection of the case*	
− Suggestions made by any team member and decision made by the team	− Suggestions can be made by any team member; decision is mostly made by the team based on observed difficulties
	− DSCC is repeated in case of failed interventions
*Handling of the IdA in preparation for the DSCC*	
− Use of the IdA to outline, at minimum, the problem to be discussed and expectations for the DSCC	− Processing of all IdA domains (a-e) in advance and pre-selection of domains for the behaviour analysis, both performed by the case reporter
*Additional information*	
− Collection and preparation of background information on the resident	− Collection and preparation of biographical and medical background information on the resident
*Handling of the IdA in the DSCC*	
− Formulation of hypotheses after the discussion of each domain	− Formulation of hypotheses after the discussion of each domain, in part and only at one point after the discussion of all domains
− Use of all five domains	− Use of selected domains; the number depends on the case characteristics and time frame of the DSCC
*Dissemination of information*	
− Dissemination of information about the results of the DSCC to entire nursing team and documentation of results in the nursing record	− Dissemination of information about the results of the DSCC to the entire nursing team through verbal briefing, flip chart documentation, written minutes and/or the intranet and documentation of results in the nursing record; difficulty of disseminating information to night and part-time staff
*Delivery of the care interventions*	
− Integration of hypothesis-driven care intervention into nursing practice after the DSCC	− Integration of hypothesis-driven care intervention into nursing practice after the DSCC
	− Difficulty of defining reasonable time frames for the delivery of interventions and of ensuring timely delivery in case of multiple interventions
	− Inhibiting factors: involvement of external people, vacation time, and overall high workload
	− Promoting factors: defined responsibility and defined time limit
*Evaluation of the care interventions*	
− Evaluation at the beginning of the subsequent DSCC	− Shifting evaluation to team meetings due to a lack of DSCC participant continuity
	− Partly visual evaluation system suitable for prompt and ongoing evaluation

Key

WELCOME-IdA Wittener model of case conferences for people with dementia, *DSCC* dementia-specific case conference, *IdA* Innovative dementia-oriented Assessment System

### Response to role structure

#### Moderator

People without leadership functions (E29, E79) sometimes undertook the role of the moderator. This approach was welcomed by nursing staff because participants tended to stay silent during the DSCC when the head ward nurse guided it. Role conflicts also occurred when the moderator was the primary caregiver of the resident being discussed. One participant reported, *“[...] last time, the moderator was the primary nurse of the resident, and he felt the need to step out of the moderator’s role and to bring in his own suggestions, his own remarks” (E75, R2_WB1, 26–27).* For this reason, some nursing homes always selected the moderator from a different nursing ward (*external moderator*) (E29, E75).

The moderators themselves reported several difficulties that they had to overcome at the beginning of the use of WELCOME-IdA. For instance, they had to manage many “sideshows” (e.g., whispering, a lack of concentration) and were confronted with large groups (> 10 persons), with showing off and rivalry among some colleagues and shyness among others (E29). It also proved difficult for moderators to set aside their personal feelings and assert their authority: *“Sometimes you also have feelings of resentment towards individual colleagues, and while you still have to fulfil your own role as a moderator, and then there are colleagues sitting there you don’t get along with so well, and you are still not feeling confident; then, it is difficult. [...] Then, especially as a moderator, you may not dare to put this person in their place” (E29_ZI_Mod_114).* For these situations, the trainers introduced a *common sign* during the on-the-job trainings that enabled the moderator to briefly step out of the moderator’s role to express his or her personal opinion on a factual level. To further reduce disturbances, the *seating* of the participants was rearranged so that members of the nursing staff did not sit next to other members of their nursing team. Such arrangements were made to break up established groupings and to promote active discussion.

To further support the moderator, an additional *co-moderator* was appointed in some nursing homes. This co-moderator watched the time and assisted the moderator if necessary. In E29, all the trained moderators (*n* = 6) also participated in all of the on-the-job trainings. After every such supported DSCC, a *peer debriefing took* place with all moderators to reflect on the previous DSCC and to strengthen the moderators in their position.

In another nursing home (E75), the moderators were asked to take on all the DSCC roles during the on-the-job training to ensure that they understood each perspective. This was expected to promote mutual support in the DSCCs.

Altogether, the role of the moderator was deemed highly important and was adapted in different ways at each nursing home.

#### Case reporter

This role was predominantly performed by the primary caregiver of the resident being discussed (E29, E82). One nursing home (E75) decided that the roles of the case reporter and the keeper of the minutes should be performed by one of the *trained moderators* who was not responsible for moderating the current DSCC. This was mainly because it would ensure that at least three moderators with some experience applying the DSCC format would take part and form the core team. The replacement of the primary caregiver by a skilled moderator was possible because there were many leading ward nurses who knew their residents very well among the moderators. After the on-the-job training, the role was transferred from the trained moderators to the primary caregivers, although the primary caregivers were still supported by the moderators.

In another nursing home (E79), a pair consisting of either two registered nurses or a registered nurse and a nursing assistant served as the case reporter (*in tandem*). This practice was justified by the difficulty of assessing the resident’s behaviour single-handedly in preparation for the DSCC. In particular, selecting case relevant domains for the behaviour analysis turned out to be very difficult because the staff considered all topics equally important.

The interviewees considered the WELCOME-IdA manual, the checklist for the case reporter and the project folder promoting factors in preparation for the DSCC. Because the role of the case reporter was assigned anew for each DSCC, the checklist proved particularly beneficial (E29, E79, E75).

#### Keeper of the minutes

The results of the DSCCs were recorded in all the nursing homes during both the on-the-job trainings and the off-the-job trainings. However, the role of the keeper of the minutes was performed differently. At some of the nursing homes (E29, E79), for the purpose of time saving, the keeper of the minutes received a copy of the IdA sheets, which had been completed by the case reporter in advance. The keeper of the minutes then added or corrected information only if the participants in the DSCC disagreed with the case reporter’s assessment.

In the other nursing homes, several people took the minutes. In one nursing home (E75), the results of the DSCC were first written on a flip chart by one person and then written up by a secretary. This allowed the results to be made available electronically in a timely manner.

It was considered beneficial if the keeper of the minutes came from another team that was not responsible for the care of the resident being discussed in the DSCC. The interviewees stated that if the keepers of the minutes were too involved in the care of the resident, they quickly switched roles and did not concentrate on taking the minutes of the DSCC.

#### Role of reflection partners

Departing from the WELCOME-IdA, the reflection partners consisted of nursing staff from different nursing wards. In particular, the steering groups reported advantages of mixing nursing staff from different nursing wards. From their point of view, the nursing staff got to know new residents, exchange among the nursing teams took place, and staff “*got to know each other in the first place*” (E29_ZI_SG, 129). According to the steering groups, the contributions of the people who did not know the resident provided different perspectives and/or raised important objections: “[...] *there emerged incredibly good ideas because we are slowly realizing that the tunnel vision has vanished; you are simply routine-blinded, and that gets opened up. That’s just a great thing [ …]*” *(E29_ZI_SG, 129–131).*

The nursing staff had opposing views of the mixing of nursing staff from different wards. Although some nursing staff supported the argumentation of the steering groups, the integration of people from other nursing wards was also described as inhibiting. Staff members felt embarrassed, and they did not want to expose their colleagues. Furthermore, they expressed that it was difficult to accept the contributions of others without commenting. In one case, a person from a different team was so dominating and at the same time not familiar enough with the case to identify the main issue that the goal of the DSCC was not accomplished, according to an interviewee: “[...] *because of one colleague who works for a different nursing ward and who was so dominating, presenting her opinion, everybody just fell into line with her, and the actual problem wasn’t identified at all. Afterwards, we simply repeated the case conference within the original team constellation, [...] and then we got on the right track” (E29_ZI_WB1, 115).* It was also seen as difficult for nursing staff from other nursing wards to assess the problem of the resident and the organizational structure of the resident’s nursing ward. “*One tends to say little. [ …]* *Here, one is dependent on what the nursing staff of the other nursing ward says*” *(E29_ZI_WB2, 84–86).* Among the nursing staff, their predominant view was that the DSCCs increased and improved communication within their own team rather than between different teams.

In addition, in some nursing homes, there were differences in intra-professional approaches in terms of the social service staff also participating in some DSCCs (E29, E79). This was viewed as an advantage by the nursing staff as the social service staff added a different perspective, and the nursing staff benefited from their comments. Furthermore, the social service staff benefited from the information discussed during the DSCC and learned to understand the nursing staff’s point of view. Knowledge was circulated in an interdisciplinary manner: *“Before, it was more like, okay, the social service staff is doing this, and the nurses are doing that. We actually didn’t know what we expected from each other. However, now, we somehow know what we are heading for with Mrs. X, and we work on that together” (E79_ZI_WB1 + 2, 126).* Moreover, team structures were strengthened, and interface problems between professions and divisions were addressed, as one steering group pointed out: *“In this home, there has been a certain kind of culture, well, two nursing wards, [ …]* *and the social service staff caught in the middle, always torn between them. [...] Now, we are doing the DSCC across our home; there is adult day care involved, there are always colleagues from other nursing wards, always someone from social service staff, and that is something people are feeling very, very positive about” (E29_ZI_SG, 105).*

### Response to group size

WELCOME-IdA recommends having 5–8 participants per DSCC, a guideline that was partially adhered to during the on-the-job training and off-the-job training. In two nursing homes (E29; E79), the groups were at least twice as large, whereas in one nursing home (E82), the minimum recommended group size was typically not achieved (< 5 persons). The expansion of the number of participants was explained by the fact that nursing staff whose nursing wards did not participate in the study and nursing staff from the day care centre were specifically invited to join the DSCCs (E29). The idea was to include as many people as possible to be able to compensate for absent staff in future. Thus, no staff members were excluded: *“We didn’t exclude anyone, you know; the moderators were designated, and the rest of the team then made up the core team. Nobody was left out” (E79_ZI_SG, 58).* The integration of staff members who were not defined as the study population resulted in different levels of knowledge about the execution of the DSCCs and the application of the IdA at the beginning of the on-the-job training phase (E29_R4_WB2, 29–32). The large group sizes also made the moderators and case reporters feel uncomfortable in their roles, and non-targeted discussions arose. As a result, attempts were made to reduce the group size. On the other hand, a group size smaller than 5 was perceived as too small because several reflection partners are needed in addition to the moderator, the case reporter and the keeper of the minutes.

### Response to core nursing teams

As indicated earlier, there was no continuous “core nursing team”, as recommended in WELCOME-IdA, in any of the four participating nursing homes. Below are the reasons why continuity of participants was not achieved within the nursing teams:

- The group of participants involved in the DSCC was randomly composed of people who were on duty that day, as one moderator reported: *“Actually, we decided not to build core teams. […]* *Those who were there took part in the DSCC. Those who were not there did not take part” (E75_ZI_Mod_138).*

Thus, in one nursing home, the DSCC was always planned for the day on which most of the staff of the respective nursing ward was present (E79).

(b) In two nursing homes (E29, E82), the declared goal was that every staff member should participate in the DSCC. It was argued, *“I think it’s a good thing when everybody gains some insight into it and not only a certain group of people, so that everybody is somehow informed about what is going on and that the staff’s acceptance is higher. Otherwise, they feel excluded, [....] and they think to themselves, ‘Why should I also work on that [measure] all of a sudden?’” (E82_ZI_SG_96–99).* One nursing home (E29) kept a list of DSCC participants. Consequently, at the end of the intervention phase, every staff member had taken part in at least one DSCC.
(c) Due to the small size of another nursing home (E82), not just the staff of the resident’s nursing ward but the staff of the entire nursing home took part in the DSCC since everyone knew every resident.
(d) A lack of continuity with respect to participants also occurred unintentionally due to vacation, illness and part-time employment.
(e) Only moderators participated continuously. In one nursing home (E75), it was established that three moderators who were familiar with the DSCC always participated, and they formed the core nursing team.

Altogether, two reasons influenced the decision to not pursue the establishment of core nursing teams: (1) organizational factors; (2) a decision by some of the steering groups to involve as much of the staff as possible.

### Response to process structure

#### Formal preparation for the DSCC

In the course of applying WELCOME-IdA, meeting times for the DSCC were planned in advance and noted in the duty roster. During these on-the-job trainings, the educational institution of the project team determined the dates for the DSCC. After this phase, meetings were planned independently by the nursing homes for off-the-job training. During the on-the-job training phase, the meeting times were communicated 1–2 months before the DSCC took place; in the subsequent phase, the meetings were planned 1 month in advance with regard to the duty roster. The early announcement of the DSCC (1 month in advance) was necessary due to the workload involved in preparing for and organizing a DSCC.

Good preparation was considered necessary for completing the DSCC within the predefined 90-min timeframe: *“[…]* *without this preparation time, we would never be able to be done in 90 minutes” (E79_ZI_SG, 197)*. In this nursing home (E79), appointed staff members (nursing students and RNs) continued to work on the ward. This allowed the DSCC to take place with no disturbances.

#### Selection of the case

In the majority of the nursing homes, the case was selected by the nursing teams of the participating wards. In E79, monthly team meetings were used for case selection; in E82, the daily nursing handovers were used for this purpose. The main criteria for selecting a certain resident for a DSCC were perceived challenging behaviour and pressing difficulties experienced by the team in dealing with that behaviour. One team member described the selection process as follows: *“We observe the resident and identify, for example, agitation or dissatisfaction or less communication of the resident. For us this is a signal, a signal that we need to talk about this resident. That’s our starting point. During our daily handover, we bring this up and decide it’s time to conduct a DSCC for this particular resident” (E79_ZI_WB2, 15).* Sometimes a resident was selected a second time when the nurses determined that previously initiated interventions had failed. The aim of the second DSCC was to re-open the “case” and to reflect again on the reasons for the resident’s challenging behaviour to identify what needed to be changed and to generate new care interventions.

#### Handling of the IdA in preparation for the DSCC

In the first nursing home in which WELCOME-IdA was implemented (E29), the IdA was initially only completed by the entire group during the DSCC. This led to a lively exchange about the resident among the members of the nursing team, but it was very time-consuming. The full process structure of WELCOME-IdA was not completed, and the DSCC ended without any planning of care interventions (E29_R2_WB2, 14–15). Thus, a second meeting was held to complete the DSCC (E29_ZI_WB1, 19–26). Subsequently, this procedure was not continued (E29_R2_WB2, 7–10). Beginning with the third on-the-job training, all the IdA domains (a-e) were processed by the case reporter in advance of the DSCC. The case reporter conducted the assessment of the behaviour, preselected domains for the behaviour analysis and presented them at the beginning of the DSCC (E29_R2_WB2, 7–10). Consequently, a complete run-through of the WELCOME-IdA process structure was possible within the 90 min timeframe: *“And that has been very effective because we now completed [the case conference] for the first time. [...] We were getting into the discussion quite well, and for the first time, we managed to complete it insofar as we not only got the collecting of ideas and the hypotheses done but could also define interventions” (E29_R2_WB2, 7–10; 58–59).*

This change in process structure was adopted by the trainers and applied in the other nursing homes.

#### Additional information

In addition to filling out the IdA in preparation for the DSCC, the nursing teams considered the resident’s biography more intensively. In E29, a written summary of biographical and medical data was prepared and presented in the DSCC. In other nursing homes, information from external parties, such as relatives or physicians, was also obtained in advance: *“And when we individually complete the sheets in advance, we necessarily have to approach the relatives or the doctors in order to add some information or to better depict the state of affairs” (E79_ZI_SG, 193, 204–209).*

#### Handling of the IdA in the DSCC

Due to the changes in the WELCOME-IdA process with respect to preparing for the DSCC, the phases were mostly adhered to in subsequent DSCCs during the on-the-job training and off-the-job training phases in all nursing homes. One nursing home (E75) simplified the formation of hypotheses in WELCOME-IdA. Instead of forming a hypothesis after each domain of the behaviour analysis, a hypothesis was formed after taking account of all the selected IdA domains in the behaviour analysis.

The number of selected domains for behaviour analysis varied depending on the case (the resident with challenging behaviour) (E75_WB2_R4, 76–79). Additionally, the time available for the DSCC determined the number of domains discussed. In one nursing home (E75), the time slot after the on-the-job training was 1 h. Thus, only one domain was discussed because this alone took 20–30 min. The assessment of the behaviour was carried out more quickly because the case reporter had already worked on it in preparation for the DSCC (E75_WB1_R485–88). In another nursing home, which reserved a time slot of 90 min, 2–3 domains were processed per DSCC (E79_R6_WB1, 20–23) (E79_ZI_WB1, 9–10).

#### Dissemination of information

WELCOME-IdA does not provide details on how to disseminate the results of the case conference; however, defining a dissemination strategy turned out to be important for the nursing homes. Different communication channels were chosen by the nursing homes to pass the information from the DSCC on to the entire nursing team. First, the results and planned care interventions were passed on orally during subsequent nursing handovers. Some of the nursing teams used the flip chart documentation that had previously been completed during the DSCC and hung it up in the staff room. Particularly for staff who did not read the minutes of the DSCC, the flip chart method was considered clear and directly accessible. In one nursing home (E75), the results were passed on in team meetings, and the minutes of the DSCC were discussed within the nursing team. The agreed-upon plan was then posted on the intranet. Accordingly, the delivery of the planned care interventions also worked well on those nursing wards where a team meeting took place soon after the DSCC. If the team meeting took place later, the introduction of the care interventions was also delayed.

In nursing homes working with an electronic care documentation system, information about the DSCC was passed on via software programs such as Outlook® or Senso® (E29, E75, E82), which were seen as an important resource. In addition, the minutes of the DSCC were printed out in some nursing homes and filed in the project folder for reference (E29, E79). In the majority of nursing homes, there was an obligation to read and countersign the minutes (E29, E79, E75). According to a head ward nurse, the information delivery functioned “quite well” for permanent and full-time staff. It was more difficult to keep track of the level of information provided to part-time workers and night shift nurses: *“Some difficulties occurred with the nurses who work during the night shift because they are really somehow left out. Even if you explained it, they are not in touch with the FallDem thing. That was quite striking. [...] With part-time workers, it’s more difficult as well” (E79_R2_WB2_49).*

#### Delivery of the care interventions

In WELCOME-IdA, there is no general target defined regarding the time frame of delivery, but it is implied that following the DSCC, the hypothesis-driven care intervention should be integrated into nursing practice as soon as possible. During the DSCC, the participants defined a timeframe for every single intervention. It was also noted in retrospect that it was difficult to define a reasonable time frame for delivery of the identified interventions. Especially when there were several case conferences in quick succession on one ward during the on-the-job training phase, the leading ward nurse felt pressured to ensure timely delivery: *“We also realized that we had scheduled some things on short notice. Well, if you have three case conferences on your ward, that is so much that you simply can’t accomplish it as quickly as you plan to out of a feeling of euphoria during the case conference. Daily routine, then, shows that it is difficult to work through three case conferences, so to speak” (E75_R2_WB2, 57).*

Nursing staff reported various factors that delayed the delivery of the care interventions. Delays became apparent in care interventions involving external people (E79_R2_WB2, 32, 34–35), such as those involving biographical interviews with relatives or a change in medication by physicians (E29_R4_WB2, 39–43; 69–71) (E29_R6_WB2, 23–24). Likewise, time off taken by nursing staff and physicians further delayed the prompt delivery of care interventions, and sometimes the responsible person forgot about the delivery of the care intervention after taking vacation (E29_R4_WB1, 33–37). The high workload was described as an additional obstacle to the timely and continuous delivery of care interventions (E29_R4_WB1_32) (E79_ZI_WB2, 101–116; 174–179; 232–240).

Directly defining responsibilities for the delivery of the care interventions as well as the date by which the care interventions should be evaluated were found to be helpful (E79_R2_WB1, 52–53) (E79_R6_WB1, 43). In E79 and E82, the primary nurse was responsible for the realization of the care intervention. In E75, it was a registered nurse, but not necessarily the primary nurse; this was the case, for example, if another nurse was deemed better suited for performing a task such as talking to the doctors. At the same time, the nursing manager kept a supervising role, and tasks such as talking to relatives were assigned to this leader.

#### Evaluation of the care intervention

WELCOME-IdA includes an evaluation of the discussed case, including the agreed-upon care interventions, at the beginning of the subsequent DSCC. This evaluation was not performed in all nursing homes, with one reason being the lack of participant continuity (E82_ZI_SG, 44–45).

In E75, the DSCC alternated monthly between the two nursing wards during the off-the-job training phase; therefore, the team had to wait 3 months to evaluate the last case, which was considered too long (E75_R4_WB1_29–55). Specifically, it was deemed reasonable to evaluate only the care interventions for the residents in one’s own nursing ward (E75_ZI_Mod, 61–69). Hence, in E29 and E75, participants from other nursing wards took part in the DSCC, but the evaluation took place exclusively with staff from the resident’s nursing ward. While in E82, the evaluation of the case and the care interventions took place during the nursing handover, in E75, it took place during the monthly team meeting, in which almost all of the staff members of the nursing ward participated. In E75, it was considered important that the whole team participated in the evaluation and that everyone “*pulled together*” (E75_ZI_WB2, 123–124). In contrast, in E79, the evaluation of the care interventions took place in “small teams”, and not everyone had to be present (E79_R6_WB1, 43). Staff members who did not participate in the evaluation could read the results of the evaluation in the minutes, which were stored in a central location but were only read by some of the staff (E79_R6_WB1, 43).

To evaluate the care interventions, flip chart documentation was used in one nursing home (E79), and it was supplemented with a self-developed evaluation system with which the team members visualized the success or failure of each intervention: *“I implemented this additional key. It’s quite simple: a plus means it has worked, a minus means it hasn’t. They have to write a plus or a minus behind the intervention” (E79_R4_WB2, 59).*

This method also ensured that the care interventions were evaluated promptly and that evaluations were not forgotten. The primary care nurse, who was ultimately responsible for transferring the care interventions into nursing care planning, also received an overview (E29_R4_WB2, 48–68) (E79_R6_WB2, 45–48).

## Discussion

The aim of this paper was to describe the process of applying and integrating the WELCOME-IdA DSCC concept in four nursing homes and to explore the response of the nursing homes to its key elements.

### Response to role structure

The results show that the selection of staff members for the predefined roles should not be based solely on competence and time resources and that possible role conflicts should also be considered.

Based on our data, we suggest filling the more formal role of the moderator with staff members without a leadership role to prevent other participants from feeling inhibited. The exertion of structural power is still very much alive in nursing, and staff members experience mixed feelings when a manager becomes deeply involved in the preparation of a case and in reflection on practice [34]. Moreover, the role of the moderator should be performed by staff members who are not the primary caregiver of the resident and who are external to the resident’s ward to prevent the moderator from stepping out of this role and engaging in the discussion about the resident, which is in line with previous research on DSCCs [10].

The role of and the tasks undertaken by the case reporter proved particularly difficult, and the nursing homes reported substantial problems preparing the case for a DSCC, which might be partly because the nursing homes did not establish core nursing teams and thus disrupted continuous learning. Moreover, the role of the case reporter switched between the different primary care nurses from one DSCC to another. Thus, each primary care nurse requires time to establish a routine to perform as a case reporter. The different strategies applied by the nursing homes to overcome the difficulties of the case reporter in preparing the case (e.g., creating pairs, gaining support from moderators) could be included in the future implementation of DSCCs as exemplary methods. The results also reveal a gap, however, because specific training for case reporters is not yet provided. Contrary to the assumption, a structured knowledge circulation process across the educational and preparation phases with different intensities of facilitation cannot be taken for granted. Because the case reporter presents the case and therefore introduces the perspective by which the case is viewed, it is important to ensure hermeneutically trained consciousness from the start and to train the case reporter how to express an understanding of the case [35]. Thus, training of the case reporter should be required in the future curriculum.

Selecting the reflection partners solely from staff members within the resident’s ward or mixing staff members from different wards can have advantages as well as disadvantages. Cooperation and mutual understanding within the care teams can be strengthened but can also lead to rivalry over who has better knowledge of the resident and/or greater ability to reflect. This experience may stimulate withdrawal from engagement because this feeling can lead to the assumption that humiliation may occur, which undermines the conditions for constructive learning while reflecting on a case [36]. A decisive factor might be how different actors have already worked together in the nursing homes before the implementation of WELCOME-IdA. This is similar to the results of a previous study [10] in which the participation of external staff was also perceived as a hurdle when participants did not work together in daily practice. Therefore, in future implementations, the existing working structure should be addressed at the beginning of the introduction of DSCCs with the steering group. The involvement of external parties should only take place when a sufficient routine for performing DSCCs has been established in the resident’s own nursing team. This ensures sufficient competence of the moderator to address a greater heterogeneity of participants.

### Response to core nursing teams

The results indicate that WELCOME-IdA’s requirement of building core teams was not practicable for various reasons, and positive effects of mixing participants were observed. The selection of individuals for a case conference should therefore be guided by the need to achieve a match between the case and the potential participants in terms of reflection potential and solution-finding potential. Such adjustments to the intervention would provide nursing homes with greater flexibility in dealing with situations such as employee sick leave or vacation. Nevertheless, participants need to have a certain level of knowledge about WELCOME-IdA and the selected case. The particular advantages of the variability versus continuity of the participants must be weighed carefully. The moderators could have an intervening function in this process because they act as consistent key individuals who would potentially be able to counterbalance knowledge differences within the groups.

### Response to the process structure

The results indicate that the preparation and post-processing phases of WELCOME-IdA are particularly important for the success of DSCCs. At the same time, these phases show the greatest need for adaptation within the process structure of WELCOME-IdA.

In the preparation phase, the adaptation of the completion of the IdA sheets beforehand accentuates the need for knowledge circulation between staff members and third parties, such as relatives and physicians. Knowledge about the case is collected and consolidated by the case reporter, whose role is strengthened through this adaption. However, the underlying idea of the WELCOME-IdA concept is to gain a comprehensive picture by integrating the diverse perspectives of participants. The prepared analysis should provide the starting point for thorough on-site discussion and reflection on the case. Furthermore, participants should have the opportunity to propose IdA domains other than those proposed by the case reporter for an in-depth behaviour analysis.

In the post-processing phase, a crucial step for ensuring that the DSCC is successful is the circulation of results (hypotheses and identified interventions) to the entire team. The results indicate that the nursing homes chose communication modes that corresponded with the usual practices in their organization. On the one hand, adjusting modes of knowledge circulation to the social system is necessary to promote implementation [37]. On the other hand, regardless of peculiarities, research shows that some formalization of communication is also necessary [38]. In particular, the promptness and extent of information transfer are of central importance because every delay and every gap during this phase jeopardizes the implementation of the developed interventions.

Digital communication media could support the rapid dissemination of information, and visualization in the wardroom has proven to be promising. Both variants would also enable part-time employees and nursing staff on night shift to access information promptly. In addition, absences of nursing staff or other external people (e.g., physician) should be taken into account when allocating responsibilities for interventions. Moreover, the time interval between DSCCs could be scheduled differently to ensure that the responsible person has enough time to integrate the hypotheses and intervention into care planning or to consciously delegate this task.

The case-specific knowledge circulation process ended when the case was evaluated. The concept’s target of performing the evaluation at the beginning of the next DSCC did not prove viable because of incompatibility with the social system and the care routines [39]. Indeed, mixed-participant groups are not suitable for evaluating the outcomes of a case in which they were not involved. Therefore, future evaluations should take place internally, within the ward and with case-relevant staff. Team meetings or handovers have proven to be a way to evaluate the DSCC within the team. The visualization of the interventions in the wardroom combined with the request to indicate the success or failure of an intervention on the flipchart can also increase awareness of an evaluation and serve as a basis for discussion during the final evaluation of a DSCC within the team.

### Practical implications/update of the manual for conducting the DSCC

- The WELCOME-IdA intervention requires a considerable amount of time for training and facilitated practice; however, the results show that the intensive training of nursing staff is both necessary and beneficial in the long run.
- The education of nursing staff, particularly in the field of dementia care, should cover hermeneutic methods of understanding behaviour, such as those underlying the IdA. One part of the intensive training within WELCOME-IdA aims at building specific competencies. Thus, the intensification of hermeneutic methods training in basic nursing education (e.g., for both RNs and CNAs) would have an impact on the educational requirements of interventions that focus on complex reflection and understanding, such as WELCOME-IdA.

### Update the role structure/personal continuity

- Additional guidance for the preparation and post-processing of a DSCC could strengthen the role of the case reporter; more on-the-job training opportunities for case reporters are needed.
- Reflection partners: the extent to which non-staff members are integrated into the DSCC and the time point at which such integration should occur depend on the actual experience of the nursing team. In particular, inexperienced teams need sufficient time to prepare, test and practice the use of WELCOME-IdA within their teams.
- The involvement of external people is recommended when nursing teams feel experienced with conducting DSCCs. The definition of external participants depends on the organizational structure of the nursing home. Furthermore, the DSCC promotes inter- and intra-disciplinary interaction, but this interaction needs to be implemented carefully.
- The implementation of WELCOME-IdA requires personnel continuity, which could be managed by the moderator and would provide more flexibility in the organization of teams for DSCCs.
- The moderator should not be a person in a management position.

### Update the process structure

- The WELCOME-IdA process requires modifications to incorporate preparation for the DSCC. To complete a DSCC within a 60- to 90-min timeframe, an initial description of the resident’s behaviour should be developed prior to the DSCC. The pre-selection by the case reporter of IdA domains for behavioural analysis is also recommended. However, both of these adaptations of WELCOME-IdA presuppose that the team can make adaptations to both the behaviour description and domain selection in the DSCC.
- The evaluation of the DSCC needs to be reorganized because ensuring the continuous participation of reflection partners is a major challenge. It is important that the team that developed the hypotheses, defined the intervention and took care of the resident is responsible for evaluating the outcome. It is necessary for the evaluation to take place in a structured manner and to be anchored in the organization as a central component of the DSCC.
- The WELCOME-IdA manual needs to provide more details regarding behaviour description, the selection of possible domains for behaviour analysis in IdA, the dissemination of the hypotheses and interventions detailed in the DSCC to the team, and the integration of the results of the DSCC into care planning.

## Limitations

No case-specific data analysis was performed, which would have allowed for a longitudinal perspective on the implementation processes in each nursing home. Such an analysis would have allowed closer process monitoring during the implementation of the intervention, but it was not possible due to the high resource requirements of the stepped-wedge cluster RCT method chosen for testing the effectiveness of WELCOME-IdA [14]. For future studies, it would also be expedient to collect process data even after the end of the intervention phase to analyse the sustainability of the intervention and its implementation strategy. The steering group in each nursing home was also in charge of assessing the strengths and weaknesses of its organization in relation to the context in which the DSCCs were implemented. Based on the results of this assessment, a tailored implementation plan was supposed to be developed for each nursing home [20], but the data from the implementation plans were not systematically assessed and analysed within this study. Future research might also use such assessments for a more in-depth analysis of the implementation processes within each cluster.

## Conclusion

The nursing homes adopted the roles of moderator, case reporter, keeper of the minutes and reflection partners in WELCOME-IdA, though which staff member filled each role differed. The role of the moderator proved to be challenging but crucial for conducting the DSCCs and for implementation and was adapted in various ways. Likewise, the role of the case reporter proved to be important and challenging. In particular, the selection of IdA domains relevant to the case was considered difficult. Regarding the reflection partners, interviewees differed regarding whether to mix nursing staff from different wards: steering groups emphasized the benefits whereas staff members focused on the challenges. The group size of 5–8 participants per case conference was partially adopted. Some nursing homes pursued the goal of involving as many staff members as possible, while one nursing home had difficulty reaching the minimum. The element of core nursing teams who participated continuously in all case conferences was not adopted at all. Instead, there was a high level of rotation among staff members. The pre-defined process structure of WELCOME-IdA was adapted in that the assessment of the residents’ behaviour and the selection of the domains for the behaviour analysis were conducted prior to the DSCC. The evaluation of the interventions was also handled differently.

In conclusion, WELCOME-IdA does need further development. The selection and training of the moderators is a decisive element, and the moderator’s role should be reflected on throughout the process. The role of the case reporter is more challenging than assumed. Therefore, additional training should be provided for this task. The idea of building core teams seems to not be practicable. Instead, the selection of the DSCC participants can be guided by the need to achieve a match between the case and the participants in terms of reflection and solution-finding potential. WELCOME-IdA should allow more possibilities for tailored adaptations than it currently does.

## Supplementary information

**Additional file 1:** Consolidated criteria for reporting qualitative studies (COREQ): 32-item checklist.

**Additional file 2:** Innovative dementia-oriented Assessment system (IdA).

**Additional file 3:** Description of the code book.

## Data Availability

The datasets generated and analysed during the current study are not publicly available due to the legal regulations of the German Center for Neurodegenerative Diseases.

## Abbreviations

CFIR: Consolidated Framework for Implementation Research
COREQ: Consolidated Criteria for Reporting Qualitative Research
DSCC: Dementia-specific case conference
IdA: Innovative dementia-oriented Assessment System
NDB model: Need-driven dementia-compromised behaviour model
RCT: Randomized controlled trial
WELCOME-IdA: Wittener model of case conferences for people with dementia

## References

[1] Selbaek G, Engedal K, Bergh S (2013). The prevalence and course of neuropsychiatric symptoms in nursing home patients with dementia: a systematic review. J Am Med Dir Assoc.

[2] Zuidema SU, Derksen E, Verhey FR, Koopmans RT (2007). Prevalence of neuropsychiatric symptoms in a large sample of Dutch nursing home patients with dementia. Int J Geriatr Psychiatry.

[3] Zwijsen SA, Gerritsen DL, Eefsting JA, Hertogh CM, Pot AM, Smalbrugge M (2014). The development of the grip on challenging behaviour dementia care programme. Int J Palliat Nurs.

[4] Klapwijk MS, Caljouw MA, Pieper MJ, van der Steen JT, Achterberg WP (2016). Characteristics associated with quality of life in long-term care residents with dementia: a cross-sectional study. Dement Geriatr Cogn Disord.

[5] Kuronen M, Kautiainen H, Karppi P, Hartikainen S, Koponen H (2016). Antipsychotic drug use and associations with neuropsychiatric symptoms in persons with impaired cognition: a cross-sectional study. Nord J Psychiatry.

[6] Schmidt SG, Dichter MN, Palm R, Hasselhorn HM (2012). Distress experienced by nurses in response to the challenging behaviour of residents - evidence from German nursing homes. J Clin Nurs.

[7] Hazelhof TJ, Schoonhoven L, van Gaal BG, Koopmans RT, Gerritsen DL (2016). Nursing staff stress from challenging behaviour of residents with dementia: a concept analysis. Int Nurs Rev.

[8] Moniz Cook ED, Swift K, James I, Malouf R, De Vugt M, Verhey F (2012). Functional analysis-based interventions for challenging behaviour in dementia. Cochrane Database Syst Rev.

[9] Holle Daniela, Halek Margareta, Holle Bernhard, Pinkert Christiane (2016). Individualized formulation-led interventions for analyzing and managing challenging behavior of people with dementia – an integrative review. Aging & Mental Health.

[10] Holle D, Kruger C, Halek M, Sirsch E, Bartholomeyczik S (2015). Experiences of nursing staff using dementia-specific case conferences in nursing homes. Am J Alzheimers Dis Other Dement.

[11] Lichtwarck Bjørn, Myhre Janne, Goyal Alka R., Rokstad Anne Marie Mork, Selbaek Geir, Kirkevold Øyvind, Bergh Sverre (2018). Experiences of nursing home staff using the targeted interdisciplinary model for evaluation and treatment of neuropsychiatric symptoms (TIME) – a qualitative study. Aging & Mental Health.

[12] Buscher I, Reuther S, Holle D, Bartholomeyczik S, Vollmar HC, Halek M (2012). Das kollektive Lernen in Fallbesprechungen. Theoretische Ansätze zur Reduktion herausfordernden Verhaltens bei Menschen mit Demenz im Rahmen des Projektes FallDem. Pflegewissenschaft..

[13] Reuther Sven, Dichter Martin Nikolaus, Buscher Ines, Vollmar Horst Christian, Holle Daniela, Bartholomeyczik Sabine, Halek Margareta (2012). Case conferences as interventions dealing with the challenging behavior of people with dementia in nursing homes: a systematic review. International Psychogeriatrics.

[14] Reuther, S, Holle, D, Buscher, I, Dortmann, O, Müller, R, Bartholomeyczik, S, Halek, M. Effect evaluation of two types of dementia-specific case conferences in German nursing homes (FallDem) using a stepped-wedge design: study protocol for a randomized controlled trial. Trials. 2014;15:319. 10.1186/1745-6215-15-319. 10.1186/1745-6215-15-319 PMC4141097 25118091

[15] Moore G. F., Audrey S., Barker M., Bond L., Bonell C., Hardeman W., Moore L., O'Cathain A., Tinati T., Wight D., Baird J. (2015). Process evaluation of complex interventions: Medical Research Council guidance. BMJ.

[16] van Achterberg T (2015). How to arrive at an implementation plan. Complex interventions in health - an overview of research methods.

[17] Damschroder LJ, Aron DC, Keith RE, Kirsh SR, Alexander JA, Lowery JC (2009). Fostering implementation of health services research findings into practice: a consolidated framework for advancing implementation science. Implement Sci.

[18] Craig P, Dieppe P, Macintyre S, Michie S, Nazareth I, Petticrew M. Medical Research Council, G. Developing and evaluating complex interventions: the new Medical Research Council guidance. BMJ. 2008:a1655. 10.1136/bmj.a1655. 10.1136/bmj.a1655 PMC2769032 18824488

[19] Vernooij-Dassen M, Moniz-Cook E (2014). Raising the standard of applied dementia care research: addressing the implementation error. Aging Ment Health.

[20] Holle D, Roes M, Buscher I, Reuther S, Muller R, Halek M (2014). Process evaluation of the implementation of dementia-specific case conferences in nursing homes (FallDem): study protocol for a randomized controlled trial. Trials..

[21] Grant A, Treweek S, Dreischulte T, Foy R, Guthrie B (2013). Process evaluations for cluster-randomised trials of complex interventions: a proposed framework for design and reporting. Trials..

[22] Atkins S, Odendaal W, Leon N, Lutge E (2015). 25 Qualitative process evaluation for complex interventions. Complex Interventions in Health.

[23] Holle D, Muller-Widmer R, Reuther S, Rosier-Segschneider U, Graf R, Roes M, Halek M (2019). Process evaluation of the context, reach and recruitment of participants and delivery of dementia-specific case conferences (WELCOME-IdA) in nursing homes (FallDem): a mixed-methods study. Trials..

[24] Tong A, Sainsbury P, Craig J (2009). Consolidated criteria for reporting qualitative research (COREQ): a 32-item checklist for interviews and focus groups. Int J Qual Health Care.

[25] Halek M, Bartholomeyczik S (2012). Description of the behaviour of wandering in people with dementia living in nursing homes - a review of the literature. Scand J Caring Sci.

[26] Halek M, Holle D, Bartholomeyczik S (2017). Development and evaluation of the content validity, practicability and feasibility of the innovative dementia-oriented assessment system for challenging behaviour in residents with dementia. BMC Health Serv Res.

[27] Kolanowski AM (1999). An overview of the need-driven dementia-compromised behavior model. J Gerontol Nurs.

[28] Nolan L (2006). Caring connections with older persons with dementia in an acute hospital setting - a hermeneutic interpretation of the staff nurse's experience. Int J Older People Nursing.

[29] Statistisches Bundesamt, *Pflegestatistik 2015 - Pflege im Rahmen der Pflegeversicherung - Deutschlandergebnisse*. 2017: Wiesbaden.

[30] Bundesinteressenvertretung für alte und pflegebedürftige Menschen e. V (BIVA), *Rahmenvertrag gemäß § 75Abs.1 SGB XI zur Kurzzeitpflege und vollstationären Pflege*. 2018.

[31] Burke L, Miller M. Phone interviewing as a mean of data collections: lessons learned and practical recommendations, vol. 2: Forum Qualitative Sozialforschung; 2001. p. Art 7.

[32] Kuckartz U. Qualitative Inhaltsanalyse. Methoden, Praxis, Computerunterstützung (Grundlagentexte Methoden).2. Beltz Juventa Verlag: Weinheim; 2014.

[33] Buscher I, Reuther S, Holle D, Bartholomeyczik S, Halek M (2012). *Wittener Modell der Fallbesprechung bei Menschen mit Demenz mit Hilfe des Innovativen-demenzorientierten- Assessmentsystems- WELCOME-IdA*.

[34] Currie, K, Tolson, D,Booth, J: Helping or hindering: the role of nurse managers in the transfer of practice development learning. In. editors* J Nurs Manag. England;2007. 15 p. 585–594. 10.1111/j.1365-2834.2007.00804.x 17688563

[35] Gadamer H-G (2004). Truth and method. Revised second edition.

[36] Argyris C, Putnam R, McLain Smith D (1985). Developing New Frames of Reference. In. editors* Action in Science. Concepts, Methods, and Skills for Research and Intervention.

[37] Rogers EM (2003). Diffusion of innovations: free press.

[38] Simpson DD, Dansereau DF (2007). Assessing organizational functioning as a step toward innovation. Sci Pract Perspect.

[39] Greenhalgh T (2004). Diffusion of innovations in service organizations: systematic review and recommendations. The Milbank Quarterly.

