# Supplementary material for: Process evaluation of the response of nursing homes to the implementation of the dementia-specific case conference concept WELCOME-IdA: A qualitative study
Source: BMC Nurs. 2020 Feb 17;19:14. doi: 10.1186/s12912-020-0403-6 (PMC7026945; doi:10.1186/s12912-020-0403-6)
Supplement: Supplementary file 1 — Additional file 1: Consolidated criteria for reporting qualitative studies (COREQ): 32-item checklist. [file 12912_2020_403_MOESM1_ESM.pdf]

Additional file 1: Consolidated criteria for reporting qualitative studies (COREQ): 32-item checklist

Topic and Item No.	Guide Questions/Description	Comment/Reported on Page No.
**Domain 1: research team and reflexivity**		
Personal Characteristics		
1. Interviewer/facilitator	Which author/s conducted the interview or focus group?	Telephone interviews: RG, TQ, SR,UR and DH, p. 12<br>Focus group interviews: MR, MH, p.13
2. Credentials	What were the researcher's credentials?	DH, PhD, MScN, RN<br>ST, PhD, MA Social Sciences<br>RG, MA Public Health<br>RWM (data manager)<br>SR, PhD, MScN, RN<br>MH, PhD, MScN, RN<br>MR, PhD, MA Sociology, RN.
3. Occupation	What was their occupation at the time of the study?	DH: Research Associate<br>ST: Research Associate<br>RG: Research Associate<br>RMW: data manager<br>SR: Research Associate<br>MH: Junior professor, group leader.<br>MR: Full Professor, site leader, group leader.
4. Gender	Was the researcher male or female?	Female and male (each forename is described on the title page)
5. Experience and training	What experience or training did the researcher have?	DH, RG, ST and MR had extensive experience in face-to-face interviews. They had also supervised qualitative research.
Relationship with participants		
6. Relationship established	Was a relationship established prior to study commencement?	DH and SR were involved in the initial discussions with the management of the participating nursing homes.
7. Participant knowledge of the interviewer	What did the participants know about the researcher?	Participants knew about the researchers' affiliation.
8. Interviewer characteristics	What characteristics were reported about the interviewer/facilitator?	None.
**Domain 2: study design**		
Theoretical framework		
9. Methodological orientation and Theory	What methodological orientation was stated to underpin the study?	Directed content analysis, p. 14
Participant selection		
10. Sampling	How were participants selected?	
11. Method of approach	How were participants approached?	Telephone and face-to-face, p. 11, 13
12. Sample size	How many participants were in the study?	34 telephone interviews with 9 participants and 15 focus group interviews with 146 participants in total, p. 13
13. Non-participation	How many people refused to participate or dropped out? Reasons?	N/A.
Setting		
14. Setting of data collection	Where was the data collected?	Focus group interviews were conducted in the nursing homes, p. 13
15. Presence of non-participants	Was anyone else present besides the participants and researchers?	No.
16. Description of sample	What are the important characteristics of the sample?	See table 5: Characteristics of the interviewees in the telephone and group interviews
Data collection		
17. Interview guide	Were questions, prompts, guides	The overall interview topics were

	provided by the authors? Was it pilot tested?	reported, p. 13. The interview guide for the telephone interviews was pilot tested among colleague researchers.
18. Repeat interviews	Were repeat interviews carried out? If yes, how many?	No.
19. Audio/visual recording	Did the research use audio or visual recording to collect the data?	Data were audio recorded using a digital recorder, p. 12, 13
20. Field notes	Were field notes made during and/or after the interview or focus group?	Field notes were made during the focus group interviews, p. 13
21. Duration	What was the duration of the interviews or focus group?	Telephone interviews: 15 minutes on average<br>Focus group interviews: 40 minutes on average, p. 12,13
22. Data saturation	Was data saturation discussed?	No.
23. Transcripts returned	Were transcripts returned to participants for comment and/or correction?	No.
**Domain 3: analysis and findings**		
Data analysis		
24. Number of data coders	How many data coders coded the data?	coded by 1 (RG or DH) researcher and checked by a second researcher (DH, ST), p. 14.
25. Description of the coding tree	Did authors provide a description of the coding tree?	The coding tree is attached to the manuscript (appendix).
26. Derivation of themes	Were themes identified in advance or derived from the data?	Interviews were analyzed using deductive content analysis, although inductive categories have been developed, p. 14
27. Software	What software, if applicable, was used to manage the data?	MAXQDA 2018, p. 14.
28. Participant checking	Did participants provide feedback on the findings?	No.
Reporting		
29. Quotations presented	Were participant quotations presented to illustrate the themes / findings? Was each quotation identified?	Participant quotations were presented. Each quotation was identified by referring to the nursing home and interview number, e. g. p. 15
30. Data and findings consistent	Was there consistency between the data presented and the findings?	Yes.
31. Clarity of major themes	Were major themes clearly presented in the findings?	Yes.
32. Clarity of minor themes	Is there a description of diverse cases or discussion of minor themes?	Yes.
