# Supplementary material for: Process evaluation of the response of nursing homes to the implementation of the dementia-specific case conference concept WELCOME-IdA: A qualitative study
Source: BMC Nurs. 2020 Feb 17;19:14. doi: 10.1186/s12912-020-0403-6 (PMC7026945; doi:10.1186/s12912-020-0403-6)
Supplement: Supplementary file 3 — Additional file 3: Description of the code book. [file 12912_2020_403_MOESM3_ESM.pdf]

Additional file 3: Description of the code book

[Original German code book]

Code	Definition
**A. PREPARATION OF CASE CONFERENCE**<br>**[A. VORBEREITUNG DER FALLBESPRECHUNG]**	
**1. Content-related preparation**<br>**[1. Inhaltliche Vorbereitung]**	Code if the interviewee discusses the content-related preparation. Details are differentiated by using the sub-categories. In case of statements that at first do NOT fit into the already existing sub-categories, code with this superordinate category first and move into an inductive category later as appropriate.<br>[Code wird vergeben, wenn die inhaltliche Vorbereitung durch den Befragten thematisiert wird. Einzelheiten werden in den Subkategorien differenziert. Gibt es Aussagen, die zunächst NICHT in eine der bereits bestehenden Subkategorien zugeordnet werden können, wird die Aussage zunächst in diese übergeordnete Kategorie codiert und in einem zweiten Codierprozess ggf. in eine induktive Kategorie verschoben.]
1.1 Evaluation of previous case<br>[1.1. Evaluation letzter Fall]	Code if explicitly referring to the process sequence of evaluating the previously discussed case.<br>[Code wird verwendet, wenn es explizit um die Sequenz der Evaluation des letzten Falls geht.]
1.2 Planned selection of the case<br>[1.2 Geplante Fallauswahl]	*Code only if the selection of the case is „planned" prior to the DSCC (otherwise code „actual" selection of the case)!*<br>Leading questions:<br>▪ Who selects the case? Case reporter?<br>▪ What are the reasons for selecting a case?<br>▪ Is there any acute need for discussion and action regarding a particular resident or are cases selected already well before?<br>At which point is the case selected (during preparation)?<br><br>*[Nur codieren wenn Fall-Auswahl im Vorfeld der FB „geplant" (ansonsten "reale "Fall"-Auswahl)!*<br>Leitfragen:<br>▪ Wer trifft die „Fall"-Auswahl? Falleinbringer?<br>▪ Aus welchem Grund wird ein „Fall" ausgewählt?<br>▪ Besteht ein akuter Rede- und/oder Handlungsbedarf bei einem Bew. oder werden die „Fälle" langfristig für eine FB vorgesehen?<br>▪ Wann wird Fallauswahl getroffen (in der Vorbereitung)?]
1. 3. Preparation of the case<br>[1.3. Fall-Vorbereitung]	Leading questions:<br>▪ Does the case reporter perform the preparation of the case?<br>▪ WHO performs the preparation of the case?<br>▪ HOW is the preparation of the case realised? (facilitating/hindering factors involved?)<br>[Leitfragen:<br>▪ Übernimmt der Falleinbringer die „Fall"-Vorbreitung?<br>▪ WER übernimmt die Fallvorbereitung?<br>▪ WIE wird die Fall-Vorbereitung umgesetzt (fördernden/hemmenden Faktoren stecken hier drin)?]
1.4 Focus on challenging behaviour<br>[1.4 HerVer-Fokussiertes Thema]	Leading question:<br>▪ Do the teams focus on challenging behaviour in the preparation?<br>[Leitfrage:<br>▪ Fokussieren die Teams „Herausfordernde Verhaltensweisen" in der Vorbereitung?]
**2. Formal preparation of the DSCC**<br>**[2. Formale Vorbereitung]**	Code if the formal preparation is discussed by the interviewee. Details are differentiated by using the sub-categories.

	[Code wird vergeben, wenn die formale Vorbereitung durch den Befragten thematisiert wird. Einzelheiten werden in den Subkategorien differenziert].
2.1 Structural framework<br>[2.1 Rahmenstruktur]	Code if the structural framework is discussed by the interviewee. Details are differentiated by using the sub-categories.<br>[Code wird vergeben, wenn die Rahmenstruktur durch den Befragten thematisiert wird. Einzelheiten werden in den Subkategorien differenziert.]
2.1.1 Planned group size<br>[2.1.1 Geplante Gruppengröße]	Leading question:<br>▪ Planned group size?<br>[Leitfrage:<br>▪ Geplante Gruppengröße?]
2.1.2 Planned location<br>[2.1.2 Geplanter Ort]	Leading questions:<br>▪ Planned location?<br>▪ Is a fixed room provided/scheduled?<br>Is the room determined in advance?<br>Leitfragen:<br>▪ Geplanter Ort?<br>▪ Wird ein festgelegter Raum bereitgestellt/geplant?<br>▪ Steht der Raum im Vorfeld der FB fest?
2.1.3 Planned date and time<br>[2.1.3 Geplanter Zeitpunkt]	Leading questions:<br>▪ Planned date and time?<br>▪ When are date and time scheduled?<br>▪ When are date and time communicated towards staff?<br>▪ How is personnel planning performed regarding DSCCs?<br>[Leitfragen:<br>▪ Geplanter Zeitpunkt?<br>▪ Wann wird der FB Termin festgelegt?<br>▪ Wann wird der Termin an die Mitarbeiter kommuniziert?<br>▪ Wie gestaltet sich die Personalplanung im Rahmen der FBs?]
2.1.4 Planned interval<br>[2.1.4 Geplantes Intervall]	Leading question:<br>▪ Planned interval?<br>[Leitfrage:<br>▪ Geplantes Intervall?]
2.1.5 Planned duration<br>[2.1.5 Geplante Dauer]	Leading question:<br>▪ Planned duration of the DSCC?<br>[Leitfrage:<br>▪ Geplante Dauer der FB?[
2.2 Role structure<br>[2.2 Rollenstruktur]	Code if the interviewee discusses the role structure. Details are differentiated by using the sub-categories.<br>Leading questions:<br>▪ Who is responsible for the preparation of the DSCCs?<br>Which roles are already assigned during preparation?<br><br>[Code wird vergeben, wenn die Rollenstruktur durch den Befragten thematisiert wird. Einzelheiten werden in den Subkategorien differenziert.<br>Leitfragen:<br>▪ Wer ist für die Vorbereitung der FBs verantwortlich?<br>▪ Welche „Rollen“ werden bereits in der Vorbereitung eingeplant/vergeben?]
2.2.1 Planned core team<br>[2.2.1 Geplantes Kernteam]	Code describes WHO is participant in a DSCC. It is not necessary that the “core team” is mentioned because it is assumed that the interviewees know this term. Instead of coding “core team”, the individual roles (such as moderator) are coded if explicitly mentioned.<br>Leading question:<br>▪ Is a core team planned prior to the DSCC?<br>[Code beschreibt, WER Teilnehmer der FB sind. Es muss nicht unbedingt das "Kernteam" benannt werden, da davon

	ausgegangen wird, dass den Befragten diese Def. bekannt ist. Anstelle des "Kernteams" werden die einzelnen Rollen (wie Moderator) codiert, wenn diese explizit benannt werden.<br>Leitfrage:<br>▪ Ist ein Kernteam im Vorfeld für die FB geplant?]
2.2.2. Planned Organisers<br>[2.2.2.Geplante Organisatoren]	Code describes WHO performs the organisation of the DSCC and which interfaces are mentioned regarding organisation matters. Aspects related to e. g. the organisation of a moderator or case reporter are coded as the respective role.<br>Leading question:<br>▪ Who is responsible for the planning and organisation of the DSCCs?<br>[Code bildet ab, WER die Organisation der FB übernimmt und welche Schnittstellen es bei Orga-Angelegenheiten gibt. Aspekte die z.B. die Organisation eines Moderators oder Falleinbringers betreffen, werden unter der „Rolle“ Moderator oder Falleinbringer kodiert.<br>Leitfrage:<br>▪ Wer übernimmt die Planung/Vorbereitung bzw. Organisation der FB?]
2.2.3 Planned Moderator<br>[2.2.3 Geplanter Moderator]	Leading questions:<br>▪ Is a trained moderator (internal or external to the ward) appointed?<br>Is a rotation of moderators planned so that they can gain experience?<br>[Leitfrage:<br>▪ Ist ein ausgebildeter Moderator (aus eigenem oder fremden WB) geplant?<br>▪ Ist ein Wechsel der Moderatoren geplant, damit diese "Praxis" sammeln?]
2.2.4 Planned nursing ward manager Geplante WBL<br>[2.2.4 Geplante WBL]	Leading question:<br>▪ Is participation of the nursing ward manager (or deputy) planned?<br>[Leitfrage:<br>▪ Teilnahme der WBL/Wohngruppenleitung (o. Stellvertretung) geplant?]
2.2.5 Planned case reporter<br>[2.25 Geplanter Falleinbringer	Leading question:<br>▪ Is the case reporting primary caregiver determined in advance?<br>[Leitfrage:<br>▪ Ist falleinbringende Bezugspflegeperson in Vorbereitung festgelegt?]
2.2.6 Planned additional participants from support staff team<br>[2.2.6 Geplante weitere aus Betreuungsteam]	Leading question:<br>▪ Is participation of additional members of the support staff team planned?<br>[Leitfrage:<br>▪ Gibt es weitere Personen aus dem Betreuungsteam, die für die Teilnahme an der FB eingeplant werden?]
2.2.7 Planned additional participants<br>[2.2.7 Geplante weitere Teilnehmer]	Leading question:<br>▪ Is participation of other (external) persons planned?<br>[Leitfrage:<br>▪ Sind darüberhinausgehende (externe) Personen eingeplant?]
B. **REALISATION OF CASE CONFERENCE**<br>**[B. UMSETZUNG DER FALLBESPRECHUNG]**	
**3. Content-related realisation**<br>**[3. Inhaltliche Umsetzung]**	Code if the content-related REALISATION is discussed by the interviewee. Details are differentiated by using the sub-categories.<br>[Code wird vergeben, wenn die inhaltliche UMSETZUNG durch den Befragten thematisiert wird. Einzelheiten werden in den Subkategorien differenziert.]

3.1 Actual selection of the case<br>[3.1 Reale „Fall-Auswahl]	*Code only if the selection of the case is only made during the DSCC (otherwise code "planned" selection of the case)!*<br><br><u>Leading questions:</u><br>▪ Planned case was discussed? If not: …<br>▪ Change of case on short-notice?<br>▪ Selection of the case only during the DSCC?<br>*[Nur codieren wenn Fall-Auswahl erst in der FB erfolgt (ansonsten „geplante" „Fall"-Auswahl)!*<br><u>Leitfragen</u><br>▪ Geplanter Fall wurde besprochen? Wenn nicht:…<br>▪ Kurzfristig anderer Fall?<br>▪ Fallauswahl erst in der FB?]
3.2 Realisation of the DSCC<br>[3.2 Fall-Umsetzung]	<u>Leading questions:</u><br>▪ Was the predefined process structure adhered to?<br>▪ HOW is the DSCC realised (facilitating/hindering factors involved)?<br>▪ Course of the DSCC?<br><u>[Leitfragen</u><br>▪ Wurde der in den FB-Konzepten vorgesehene Ablauf eingehalten?<br>▪ WIE wird die Fall-Vorbereitung umgesetzt (fördernden/hemmenden Faktoren stecken hier drin)?<br>▪ Ablauf der Fallbesprechung?]
3.3 Focus on challenging behaviour<br>[3.3 HerVer fokussierte Themen]	<u>Leading question:</u><br>▪ Do the teams focus on challenging behaviour in the realisation of the DSCC?<br><u>[Leitfrage:</u><br>▪ Fokussieren die Teams „Herausfordernde Verhaltensweisen" in der Umsetzung der FB?]
**4. Formal realisation**<br>**[4. Formale Umsetzung]**	Code if the formal REALISATION is discussed by the interviewee. Details are differentiated by using the sub-categories. Formal REALISATION includes aspects of: structural framework of DSCC and of participants/role structure.<br>[Code wird vergeben, wenn die formale UMSETZUNG durch den Befragten thematisiert wird. Einzelheiten werden in den Subkategorien differenziert. Die formale UMSETZUNG umfasst Aspekte der: Rahmenstruktur der FBs. sowie der Teilnehmer/Rollenstruktur.]
4.1 Structural framework<br>[4.1. Rahmenstruktur]	Code if the structural framework is discussed by the interviewee. Details are differentiated by using the sub-categories.<br><u>Leading question:</u><br>▪ Was it possible to realise the planned structural elements?<br>[Code wird vergeben, wenn die Rahmenstruktur durch den Befragten thematisiert wird. Einzelheiten werden in den Subkategorien differenziert.<br><u>Leitfrage:</u><br>▪ Konnten die geplanten Strukturelemente umgesetzt werden?]
4.1.1 Actual group size<br>[4.1.1 Reale Gruppengröße]	<u>Leading questions:</u><br>▪ What was the actual group size?<br>▪ Was it possible to adhere to the recommended group size?<br><u>[Leitfragen:</u><br>▪ Wie sieht die „reale" Gruppengröße aus?<br>▪ Konnte die empfohlene Gruppengröße eingehalten werden?]
4.1.2 Actual location<br>[4.1.2 Realer Ort]	<u>Leading questions:</u><br>▪ What was the actual location?<br>▪ Where did the DSCC take place?

	[Leitfragen:<br>▪ Was war der „realer“ Veranstaltung- Ort?<br>▪ Wo hat die FB tatsächlich stattgefunden?]
4.1.3 Actual date and time<br>[4.1.3 Realer Zeitpunkt]	Leading questions:<br>▪ What was the actual date and time for the DSCC?<br>▪ Was it possible to realise the planned date and time?<br>▪ Has rescheduling occurred?<br>▪ Reasons for rescheduling?<br>▪ How are postponements communicated towards staff?<br>▪ How is personnel planning performed regarding the realisation of DSCCs?<br>[Leitfragen:<br>▪ Was war der „realer“ Zeitpunkt der FB?<br>▪ Konnte der geplante FB Termin umgesetzt werden?<br>▪ Gab es Terminverschiebungen?<br>▪ Gründe für die Terminverschiebung?<br>▪ Wie werden Terminveränderungen an die Mitarbeiter kommuniziert?<br>▪ Wie gestaltet sich die Personalplanung im Rahmen der FB Umsetzung?]
4.1.4 Reales Intervall<br>[4.1.4 Actual interval]	Leading questions:<br>▪ Was it possible to adhere to the planned appointments/interval?<br>▪ Which reasons/(context) factors caused that the interval could (not) be realised?<br>[Leitfragen:<br>▪ Konnten die geplanten Termine/Intervalle eingehalten werden?<br>▪ Welche Gründe/(Rahmen-)Bedingungen haben dazu geführt, dass Intervalle (nicht) eingehalten wurden?]
4.1.5 Actual duration<br>[4.1.5 Reale Dauer]	Leading questions:<br>▪ Actual duration of DSCC?<br>▪ Are there differences from the planned duration?<br>▪ Does the duration differ between individual DSCCs?<br>[Leitfragen:<br>▪ „reale“ Dauer für die FB<br>▪ Gibt es Unterschiede zur geplanten Dauer der FB?<br>▪ Unterscheidet sich die Dauer der einzelnen FBs?]
4.2 Role structure<br>[4.2 Rollenstruktur]	Code if the role structure is discussed by the interviewee. Details are differentiated by using the sub-categories.<br>Leading questions:<br>▪ Which roles are represented during a DSCC?<br>▪ Was it possible to reach the minimum number of participants and/or role keepers?<br>[Code wird vergeben, wenn die Rollenstruktur durch den Befragten thematisiert wird. Einzelheiten werden in den Subkategorien differenziert.<br>Leitfragen:<br>▪ Welche „Rollen“ sind in der FB vertreten?<br>▪ Konnte die Mindestanzahl an Teilnehmern und/oder Rollenvertretern erreicht werden?]
4.2.1 Actual core nursing team<br>[4.2.1 Reales Kernteam]	Leading questions:<br>▪ Does the planned core team participate in the DSCC?<br>▪ Which participants were absent during the scheduled DSCC and why?<br>Did overlapping between roles occur or did participants act as multiple role keepers (moderator and case reporter are the same person)?<br>[Leitfragen:<br>▪ Nimmt das geplante Kernteam an der FB teil?<br>▪ Welche Teilnehmer fehlten zur geplanten FB und warum?

	▪ Gab es Überschneidungen in den Rollen bzw. gab es mehrfache Rollenträger (Moderator und Falleinbringer ist gleiche Person)?]
4.2.2 Actual organisers<br>[4.2.2 Reale Organisatoren]	Leading questions:<br>▪ Who cares for the actual realisation of the DSCC?<br>▪ Who is responsible for the realisation of the DSCC?<br>▪ Are there different responsible persons (for preparation/realisation/post-processing) or is there one person where all information merges (e. g. quality management or interface coordinator)?<br>[Leitfragen:<br>▪ Wer sorgt für die „reale" Umsetzung der FB?<br>▪ Wer ist für die konkrete Umsetzung verantwortlich?<br>▪ Gibt es unterschiedliche Verantwortungsträger (für die Vorbereitung/ Umsetzung/ Nachbereitung) oder eine Person, bei der alle Informationen zusammenlaufen (z.B. QM oder Schnittstellenkoordinatorin)?]
4.2.3 Actual moderator<br>[4.2.3 Realer Moderator]	Leading questions:<br>▪ Is a trained moderator (internal or external to the ward) present?<br>▪ Could the appointed moderator participate in the DSCC?<br>▪ How do participants perceive the realisation of the role of the moderator?<br>[Leitfragen:<br>▪ Ist ein ausgebildeter Moderator (aus eigenem oder fremden WB) anwesend?<br>▪ Konnte der geplante Moderator an der FB teilnehmen?<br>▪ Wie nehmen die Befragten die Umsetzung der Moderatorenrolle wahr?]
4.2.4 Actual nursing ward manager<br>[4.2.4 Realer WBL]	Leading question:<br>▪ Participation of the nursing ward manager (or deputy)?<br>[Leitfrage:<br>▪ Teilnahme der WBL/Wohngruppenleitung (o. Stellvertretung)?]
4.2.5 Actual case reporter<br>[4.2.5 Realer Falleinbringer]	Leading question:<br>Was a case reporter or the planned case reporter present?<br>[Leitfrage:<br>▪ War ein Falleinbringer bzw. der geplante Falleinbringer anwesend?]
4.2.6 Actual keeper of the minutes<br>[4.2.6 Realer Protokollant]	Leading questions:<br>▪ Was a keeper of the minutes present?<br>▪ Did the keeper of the minutes take the minutes and are minutes available?<br>[Leitfrage:<br>▪ War ein Protokollant anwesend?<br>▪ Und hat dieser Protokoll geführt bzw. liegt ein Protokoll vor?]
4.2.7 Actual reflection partners<br>[4.2.7 Reale Reflexionspartner]	Leading question:<br>▪ Are one or more reflection partners present?<br>[Leitfrage:<br>▪ Ist ein oder sind mehrere Reflexionspartner anwesend?]
4.2.8 Actual additional participants from support staff team<br>[4.2.8 Reale weitere aus Betreuungsteam]	Leading question:<br>▪ Did other internal persons participate?<br>[Leitfrage:<br>▪ Haben weitere interne Personen teilgenommen?]
4.2.9 Actual additional participants<br>[4.2.9 Reale weitere Teilnehmer]	Leading question:<br>▪ Did other external persons participate?<br>[Leitfrage:<br>▪ Haben weitere externe Personen teilgenommen?]

C**. POST-PROCESSING OF DSCC<br>[C.NACHBEREITUNG DER FALLBESPRECHUNG]**	
5.1 Planning and delivery of care interventions<br>[5.1 Maßnahmenplanung/umsetzung]	Leading question:<br>▪ Were any care interventions planned and/or realised following the DSCC?<br>[Leitfrage:<br>▪ Wurden Maßnahmen geplant und/oder im Anschluss an die FB umgesetzt?]
5.2 Informing staff members<br>[5.2 Information der Mitarbeiter]	Leading questions:<br>▪ How are dissemination of information and documentation handled within the nursing ward?<br>▪ How were nursing team members informed about the results of the previous DSCC?<br>[Leitfrage:<br>▪ Wie funktionier die Informationsweitergabe und Dokumentation auf dem WB/Cluster?<br>▪ Wie wurden die Teammitglieder über die Ergebnisse aus der letzten FB informiert?]
**D. EVALUATION OF WELCOME-IdA<br>[D.BEWERTUNG WELCOME-IdA]**	
6.1 DSCC_Evaluation_Development<br>[6.1 FB_Bewertung_Entwicklungsprozess]	EVALUATION: During the interviews, the interviewees are asked about their perceptions, assessments and opinions, individual components of the INTERVENTION are evaluated. This category should include all evaluating statements of interviewees.<br>DEVELOPMENT: Furthermore, experiences, change and development within the context of DSCCs are reported.<br>In addition, REASONS for ADOPTION or ADAPTION are coded here.<br>[BEWERTUNG: In den Interviews werden die Beteiligten nach ihren Wahrnehmungen, Einschätzungen und konkreten Meinungen befragt, es kommt zu Bewertungen der einzelnen Komponenten der INTERVENTION.<br>In dieser Kategorie sollen alle „wertenden“ Aussagen der Befragten zusammengestellt werden.<br>ENTWICKLUNG: Ebenfalls werden Erfahrungen, Veränderungen und Entwicklungen im Rahmen der FBs berichtet. Auch werden hier GRÜNDE für die Übenahme (ADOPTION) oder Anpassungen (ADAPTION) zusammengetragen.]
6.2 DSCC_Trainings/ToJ<br>[6.2 FB-B_Schulungen/ToJ]	All evaluating statements regarding the trainings are coded here.<br>[Hier werden alle wertenden Aussagen zu den Schulungen erfasst.]
6.3 DSCC_Concept (IdA)<br>[6.3 FB-B_Konzept (IdA)]	Here, it becomes apparent for response of cluster which problems with using the IdA sheets occur and which were adopted/adapted by the nursing homes and which were NOT.<br>[Für Respones of Cluster wird hier deutlich, welche Probleme in der Anwendung des IdA-Bogens bestehen und welche durch die Einrichtung übernommen/angepasst oder NICHT-übernommen wurden.]
6.4 DSCC_Sequences general<br>[6.4 FB_B_Sequenzen generell]	Here, all evaluating statements regarding the general sequential course of the DSCCs are coded. Mere descriptions of the status quo, e. g. at the beginning of the focus group interview, that describe the process of conducting a DSCC are coded with DSCC_Content-related_Realistion_Case-realisation.<br>[Hier werden alle wertenden Aussagen zum generellen sequenziellen Ablauf der FBs codiert. Reine Zustandsbeschreibungen z.B. am Anfang der ZI in denen der Ablauf einer FB beschrieben wird, werden unter FB_Inhaltliche_Umsetzung_Fall-Umsetzung codiert.]

6.5 DSCC_Preparation<br><br>[6.5 FB_B_Vorbereitung]	Leading questions:<br>▪ How do participants evaluate the PREPARATION processes?<br>▪ How do they perceive the PREPARATION?<br>[Leitfragen:<br>▪ Wie bewerten die Befragten die VORBEREITUNGsprozesse auf die FB?<br>▪ Wie nehmen sie die VORBEREITUNG wahr?]
6.6 DSCC_Realisation<br><br>[6.6 FB_B_Umsetzung]	Leading questions:<br>▪ How do participants evaluate the REALISATION processes?<br>▪ How do they perceive the REALISATION?<br>▪ Continuation of project beyond the actual project phase?<br>[Leitfragen:<br>▪ Wie bewerten die Befragten die UMSETZUNGsprozesse auf die FB?<br>▪ Wie nehmen sie die UMSETZUNG wahr?<br>▪ Weiterführung des Projektes über die eigentliche Projektphase hinaus?]
6.7 DSCC_Post-processing<br>[6.7 FB_B_Nachbereitung]	Leading questions:<br>▪ How do participants evaluate POST-PROCESSING processes? How do they perceive the POST-PROCESSING?<br>▪ Evaluating statements regarding the planning of care interventions or informing staff members are coded here.<br>[Leitfragen:<br>▪ Wie bewerten die Befragten die NACHBEREITUNGsprozesse auf die FB? Wie nehmen sie die NACHBEREITUNG wahr?<br>▪ Hier werden auch "wertende" Informationen zur "Maßnahmenplanung" oder "Information der Mitarbeiter" hinterlegt.]
6.8 DSCC_Handling Challenging Behaviour<br><br>[6.8 FB-B_Umgang HerVer]	Leading question:<br>▪ Code any information with regards to the development of the handling of challenging behaviour in the course of the DSCCs.<br>[Leitfrage:<br>▪ Hier wird alles zu der Entwicklung im Rahmen der FB im Umgang mit Herausforderndem Verhalten dokumentiert]
6.9 Miscellaneous<br><br>[6.9 Sonstiges]	Category gives room for any other information regarding the INTERVENTION that is not covered by any other category. This information is hence captured and it is possible to decide within an additional round of analysis whether it can be transferred into a useful code?!<br>[Kategorie soll Raum für „Sonstige“ Informationen bezüglich der INTERVENTION lassen, die durch die anderen Kategorien nicht abgedeckt sind.<br>Diese werden somit erfasst und man kann überlegen diese bei einem weiteren Analysedurchgang als sinnvollen Code zu verwenden?!]
